# The Buffering Effect of Resilience on the Social Consequences of COVID in Older Women

**DOI:** 10.1093/geroni/igab046.3737

**Published:** 2021-12-17

**Authors:** Nacia Goldberg, Dawn Carr, Miles Taylor, Natalie Sachs-Ericsson

**Affiliations:** Florida State University, Tallahassee, Florida, United States

## Abstract

Prior to the pandemic, public health experts argued that loneliness was among the most significant threats facing women’s health and wellbeing. As the COVID-19 pandemic brought our social lives to an abrupt pause in March, 2020, older adults were encouraged to remain isolated from friends and family. Social distancing guidelines led many older people to decrease social interactions with others. Using a community-based longitudinal study of women age 60+, we examined how changes in feelings of social connections with others influenced loneliness in October 2020 relative to prior to the pandemic (in September 2018). Our previous research has shown that psychological resilience decreases the negative consequences of major life stressors in later life. We hypothesized that women with high social consequences of the pandemic would experience increased loneliness, but resilience would buffer these effects. In line with our hypotheses, results showed that those who reported significant declines in social connectedness with others during the pandemic (i.e., high social consequences) experienced significant increases in loneliness (beta=0.125; p<0.001). Resilience, alternatively, was significantly associated with decreased loneliness (beta=-0.05; p<0.05), and buffered the social consequences of the pandemic. That is, as resilience increased, the social consequences of COVID-19 significantly declined (p<0.01), and resilience attenuated the negative consequences of high levels of social consequences of COVID-19 on loneliness, while those with high social consequences and low resilience experienced significant increases in loneliness in association with the pandemic. Based on our findings, we discuss potential clinical implications for resilience-based interventions for older adults.

